# ^68^Ga-DOTATATE and ^18^F-FDG PET/CT in Paraganglioma and Pheochromocytoma: utility, patterns and heterogeneity

**DOI:** 10.1186/s40644-016-0084-2

**Published:** 2016-08-17

**Authors:** Chian A. Chang, David A. Pattison, Richard W. Tothill, Grace Kong, Tim J. Akhurst, Rodney J. Hicks, Michael S. Hofman

**Affiliations:** 1 Cancer Imaging, Peter MacCallum Cancer Centre, 305 Grattan Street, Melbourne, VIC 3000 Australia; 2 Sir Peter MacCallum Department of Oncology, University of Melbourne, Melbourne, Australia; 3 Endocrinology Service, Peter MacCallum Cancer Centre, Melbourne, Australia

**Keywords:** Ga-68 DOTATATE, F-18 FDG, PET/CT, Paraganglioma, Pheochromocytoma

## Abstract

**Background:**

Pheochromocytomas (PCC) and paragangliomas (PGL) are neuroendocrine tumours arising from pluripotent neural crest stem cells and are associated with neurons of the autonomic nervous system. PCCs/PGLs are often hereditary and multifocal, and their biologic behaviour and metabolic activity vary making imaging of these tumours challenging. The imaging gold standard has been I-123 MIBG complemented by CT or MRI. PGLs being neuroendocrine tumours express somatostatin receptors enabling imaging with Ga-68 DOTA-coupled peptides such as DOTATATE. Imaging with F-18 FDG also provides additional information regarding metabolic activity and biologic aggressiveness of these tumours, or, in some situations, reflecting metabolic reprogramming of these tumours. We report our experience using both Ga-68 DOTATATE and F-18 FDG PET/CT imaging in patients with PGLs and PCCs.

**Methods:**

This was a retrospective review of 23 patients with proven PGL/PCC who underwent both DOTATATE and FDG PET/CT. Seven patients also had I-123 MIBG SPECT/CT and 1 patient had I-124 MIBG PET/CT. Lesional intensity and patterns of uptake were analysed.

**Results:**

DOTATATE and FDG were positive at most sites of disease (96.2 % vs 91.4 %), although uptake intensity was significantly higher on DOTATATE with a median SUV of 21 compared to 12.5 for FDG (*p* < 0.001). SUVmax on F-18 FDG was significantly higher (*p* < 0.001) in clinically aggressive cases. I-123/I-124 MIBG detected fewer lesions (30.4 %).

**Conclusion:**

Overall, Ga-68 DOTATATE PET/CT detected similar number but has significantly greater lesion-to-background contrast compared to F-18 FDG PET/CT. Combined with high specificity, patient convenience and relatively low cost, DOTATATE PET/CT should be considered the ideal first line investigation for imaging PGL/PCC. Depending on DOTATATE findings and the clinical question, FDG and MIBG remain useful and, in selected cases, may provide more accurate staging, disease characterisation and guide treatment choices.

## Background

Paragangliomas (PGL) are neuroendocrine tumours arising from pluripotent neural crest stem cells and are associated with neurons of the autonomic nervous system occurring anywhere along the paravertebral axis from the base of skull to the pelvis. These can be associated with the sympathetic nervous system and derived from the adrenal medulla, organ of Zuckerkandl or other chromaffin cells that may persist beyond embryogenesis and, therefore, are more likely located in the abdomen or pelvis. PGL associated with the parasympathetic nervous system are derived from cells in the parasympathetic branchiometric paraganglia (chemoreceptors) and are mainly located in the head, neck and mediastinum. Pheochromocytoma (PCC) is reserved solely for an adrenal paraganglioma according to the 2004 WHO classification of tumours.

There is a high frequency of hereditary forms of PGLs and PCCs (25–35 %) and these in turn, may be multifocal [1, 2]. Molecular genetic research to date has so far identified multiple susceptible genes for tumours of the paraganglia system [3–5], which can be classified into two groups. The pseudohypoxic cluster is characterised by constitutive activation of hypoxia inducible factors leading to inhibition of oxidative phosphorylation and activation of glycolytic pathway via the Warburg effect – includes the succinate dehydrogenase (SDH) subunit B, C, D, complex assembly factor 2 (AF2, also called SDH5) and A, Von Hippel-Lindau disease (VHL), Hypoxia Inducible Factor (HIF) Type 2 and fumarate hydratase (FH) genes. The kinase signalling subgroup, includes characteristic mutations in REarranged during Transfection (RET) in Multiple Endocrine Neoplasia type 2 (MEN2), Neurofibromatosis type 1 (NF1), transmembrane protein 127 (TMEM127), MYC associated factor X (MAX), EGLN1 (PHD2), KIF1 and IDH1 [6]. Our group has recently described a technique for assigning tumours into these subcategories [7]. PCCs may coexist with other tumour types in MEN2A, MEN2B, VHL and NF1. Extra-adrenal PGLs may also occur in other multiple neuroendocrine syndromes such as Carney-Stratakis dyad and Carney’s triad.

Although most PGLs and PCCs are benign and slow growing, metastasis or malignant degeneration can occur. The reported rate varies but it appears that PGLs associated with the SDHB germline mutation carries a significantly higher rate of a more aggressive behaviour locally and developing metastatic disease [8, 9]. Sympathetic PGLs may hypersecrete catecholamines while parasympathetic PGLs are mostly non-secretory. Somatostatin cell surface receptor (SSR) overexpression is common in neuroendocrine tumours including PGL and PCCs. SSR type 2 is the most commonly overexpressed [10, 11].

Molecular imaging in combination with anatomic imaging is often required to stage and monitor treatment response of PGLs and PCCs and also provides information relevant to selection of radionuclide therapy in the context of metastatic disease. The historical molecular imaging gold standard is with I-123 Metaiodobenzylguanidine (MIBG) [12] combined with anatomic imaging with CT or MRI. Somatostatin receptor based imaging has also been performed with In-111 DTPA Pentetreotide SPECT/CT [13]. More recently, newer radiopharmaceuticals particularly positron emitter imaging with PET/CTs such as with F-18 FDG or Ga-68 DOTA-coupled peptides have been shown to have higher sensitivity and better imaging characteristics in the staging and restaging of this disease [14]. There are currently three DOTA-coupled peptides in clinical use (DOTATATE, DOTANOC and DOTATOC), all showing especially high affinity for somatostatin surface receptor type-2. The advantages of PET/CT versus SPECT/CT and the intricacies of SSR binding using DOTA coupled peptides versus In-111 DTPA Pentetreotide have been extensively discussed elsewhere [2, 14–19] and will not be addressed here.

We report our experience of imaging patients with PGLs and PCCs who had both Ga-68 DOTATATE and F-18 FDG PET/CT imaging. A subset of patients also had I-123 SPECT/CT or I-124 MIBG PET/CT enabling a limited comparison between the historical molecular imaging gold standard and newer molecular imaging techniques. Our aim was to evaluate the diagnostic utility and patterns of uptake on DOTATATE and FDG in the detection of PGL and PCC. Secondary aims were to compare DOTATATE and FDG to MIBG, the historical molecular imaging gold standard, assess the utility of molecular imaging in the different germline mutations, predicting aggressive disease and patterns of brown adipose tissue (BAT) uptake on ^18^F FDG related to hormone secreting tumours.

## Methods

In this retrospective study, we searched our radiology information system (RIS) for patients with PGL or PCCs who underwent both DOTATATE and FDG PET/CTs within 6 months of one another. The study period encompassed all patients imaged between August 2011 and November 2014. 34 patients were identified, of whom 23 patients (13 males, 10 females, mean age 43, range 21–74) had no changes in management in the interval between the scans and were included in this study. 14 patients had both DOTATATE and FDG PET/CTs either on the same day or 1 day apart, 4 patients had scans within 1 month and five patients had scans within 2 to 6 months. Seven patients had I-123 MIBG SPECT/CT and one patient had I-124 MIBG PET/CT as part of work-up to assess suitability for I-131 MIBG therapy. This study was approved by the institutional ethics review board. As a clinical practice audit, the need for patient consent was waived.

Six patients were imaged as part of initial work-up and staging while the remaining 15 patients had prior treatments. For 13 of these patients, the DOTATATE PET was performed, in part, to assess potential suitability for peptide receptor radionuclide therapy (PRRT). 7 patients had confirmed SDHB mutation, one patient each had SDHD, SDHA, or MEN2A, three had no germline mutation identified and seven were not tested. No adverse events were observed patients after administration of DOTATATE, FDG or MIBG.

### Image acquisition and analysis

All PET/CT images were acquired on a hybrid PET/CT device (either Biograph 64 with TrueV, Siemens Medical Systems or Discovery 690, GE Healthcare). The injected dose of DOTATATE was 3.1 MBq/kg with an uptake time of between 45 to 60 min. Patients were not required to fast for the study. The injected dose of FDG was 3.6 MBq/kg with an uptake time of 60 min. Patients were required to fast for a minimum of 6 h and radiopharmaceuticals was injected if blood sugar levels were less than 10.0 mmol/L. Planar and SPECT/CT imaging were acquired on a hybrid SPECT/CT device (Symbia T6, Siemens Medical Systems). Injected dose of MIBG was approximately 340 MBq and imaging was performed 24 h later (+/− 2 h).

Any lesion demonstrating activity higher than the physiologic uptake of the involved organ was considered a “true” lesion unless correlative anatomic imaging suggested non malignant or alternative pathology. PET/CT images were evaluated qualitatively and semiquantitatively. For lesion assessment, if there were multiple lesions in one particular organ, the 3 lesions with the highest SUVmax was recorded. Sites showing discordant uptake (increased DOTATATE uptake but no FDG uptake or vice versa) were also recorded. Qualitatively, DOTATATE uptake intensity was graded as mild, moderate or intense corresponding to faint, intensity less than, equal to, or greater than liver, respectively. The presence, intensity and distribution of BAT uptake on FDG and administration of alpha and/or beta blockers at the time of the study were also recorded. BAT uptake was graded as none, mild = present in one region (neck and supraclavicular region, thoracic paravertebral/mediastinal region, or below the diaphragm/perinephric region) (SUVmax > 2.5), moderate = present in two regions, and severe = present in all three regions. I-123 MIBG images was evaluated qualitatively both on planar and SPECT/CT. Uptake intensity was graded as none, mild = faint, moderate = less than or equal to hepatic uptake and intense = higher than hepatic uptake. The I-124 MIBG PET/CT was evaluated identically to I-123 MIBG studies.

### Statistical analysis

Lesion based sensitivities were compared between DOTATATE, FDG and MIBG. For comparison of SUVmax difference between DOTATATE and FDG, and between aggressive and non-aggressive patients for DOTATATE and FDG, the Mann–Whitney test was used.

## Results

Nineteen patients had multifocal metastatic disease and the remaining four patients had disease confined to one site of disease (three patients with disease confined to the primary site and one patient with oligometastatic disease). Fifteen patients had PGLs (14 with metastatic disease and one with disease confined to the primary site) and eight patients had PCCs (five with metastatic disease and three with non-metastatic PCC). Six patients (three with SDHB germline mutation and three with negative mutation testing) had disease progression symptomatically, biochemically and on imaging within six months despite commencement of therapy (either chemotherapy, radiotherapy, PRRT or a combination) and were classified as having an aggressive phenotype of the disease. In total, 104 lesions were identified in 23 patients and analysed and the results are summarised in Tables 1 and 2.

**Table 1:** Patient characteristics

Patient	Sex	Age	Germline Mutation	Location of Primary	Location of Metastases	Hormone Secreted
1	M	42	SDHB	R Carotid Body	Bone	Adr, NorAdr
2	M	43	SDHD	R Glomus Jugulare	Lymph node	Nil
3	M	59	SDHB	R Carotid Body	Bone, Lung	Nil
4	M	23	Negative	Retroperitoneum	Lymph node	NorAdr, Dop
5	F	31	MEN-2A	Both Adrenals	Nil	Adr, NorAdr
6	F	50	Not tested	L Adrenal	Bone, Lymph node	NorAdr
7	M	74	Not tested	R Adrenal	Nil	Adr, NorAdr
8	M	24	SDHB	Adrenal & Retroperitoneum	Bone, Lymph node	Unknown
9	F	63	Not Tested	R Glomus Vagale	Bone, Liver, Lymph node, Lung	Nil
10	M	46	SDHB	Unknown	Bone, Lymph node	NorAdr
11	F	22	Negative	L Adrenal	Bone, Lymph node	NorAdr
12	M	40	Negative	Unknown	Bone, Liver, Lymph node	Unknown
13	M	69	Not Tested	R Carotid Body	Liver, Lymph node	Nil
14	M	21	SDHB	R Adrenal & L Glomus Jugulare	Bone, Liver, Lymph node	Adr, NorAdr
15	M	35	Not Tested	Unknown	Bone, Liver, Lymph node	Nil
16	M	66	Not tested	R Carotid Body	Nil	Nil
17	M	20	Not tested	R Adrenal	Nil	Adr
18	F	26	SDHB	Retroperitoneum	Bone, Liver, Lymph node	NorAdr
19	F	49	SDHB	Pelvic	Lymph node	Nil
20	F	68	Not avail.	R Adrenal	Liver, Lymph node	Nil
21	F	32	SDHA	R Glomus Jugulo-Tympanicum	Bone	NorAdr, Dop
22	F	31	SDHB	L Carotid Body	Bone, Liver	Nil
23	F	50	Not Tested	Retroperitoneum	Nil	NorAdr

*Adr* adrenaline, *NorAdr* noradrenaline, *Dop* dopamine

**Table 2:** Number of lesions^a^ detected (%)

Lesion Site	Ga-68 DOTATATE	F-18 FDG	I-123/I-124 MIBG
Bone	41/41	37/41	3/21
Liver	14/16	16/16	3/11
Lung	5/5	5/5	1/3
Lymph Node	24/24	24/24	5/13
Primary	16/18	13/18	5/8
Total	100/104	95/104	17/56

^a^up the three lesions per organ were counted

### Ga-68 DOTATATE PET/CT findings

In 100 of the 104 (96 %) lesions analysed, DOTATATE was positive at sites of disease regardless of presence or absence of germline mutation. Semi-quantitatively, SUVmax ranged from 4 to 185 with a median of 21.0. Qualitatively, most lesions demonstrated moderate-to-high uptake intensity with intensity greater than liver. Lesions with lower intensities were generally at sites of low volume disease (smaller than 5 mm or not visible on anatomic imaging) and, therefore, best explained by partial volume effects.

**Fig. 1:**
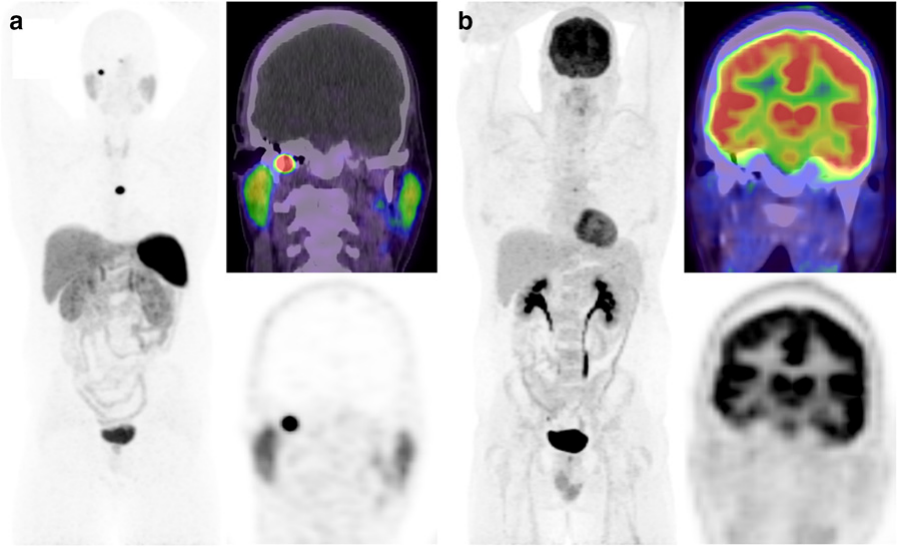
A 43 year-old male with *SDHD* germline mutation and prior excision of multiple paragangliomas. PET imaging was performed to characterise an equivocal mediastinal nodal abnormality identified on CT. Ga-68 DOTATATE (**a**) demonstrate very intense uptake in a right skull base glomus jugulare tumour (SUVmax = 90), which was not visible on F-18 FDG, in part, attributable to high uptake in physiologic cerebral activity (**b**). A left paratracheal abnormality in the mediastinum showed intense DOTATATE uptake and low grade uptake on FDG. This likely represented a further paraganglioma rather than a metastasis. The presence of 2 tumour suggests a probable germ-line mutation and would have warranted genetic testing if the germ-line mutation was not already known

**Fig. 2:**
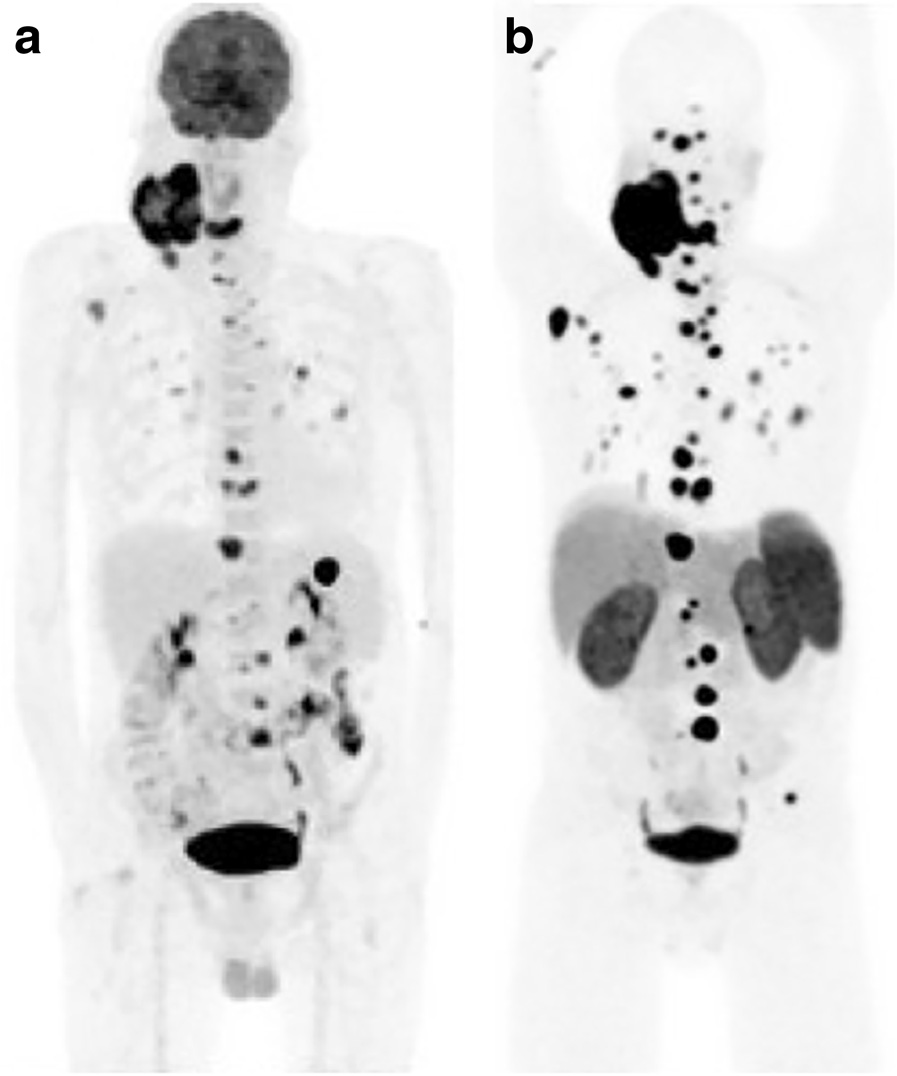
Middle-aged man with long-standing large neck carotid body tumour on the background of *SDHD*-mutation, with newly diagnosed wide-spread osseous metastatic disease on F-18 FDG (**a**). Ga-68 DOTATATE (**b**) demonstrates superior tumour-to-background contrast with identification of greater number of metastases compared to FDG. The disease stage (stage IV), however, is not altered by the greater detection rate of DOTATATE PET/CT but confirms suitability for peptide receptor radionuclide therapy

In one patient, DOTATATE PET/CT detected a glomus jugulare tumour that could not be visualised with FDG PET/CT (Fig. 1). In other patients, although additional lesions were evident on DOTATATE compared to FDG PET/CT, this did not alter stage but merely identified additional sites in patients with already known, widespread metastases (Fig. 2).

The SUVmax was lower in patients with aggressive clinical behaviour as opposed to non-aggressive cases with median of 15.5 compared to 22.0, but the difference was not statistically significantly (*p* = 0.25).

### F-18 FDG PET/CT findings

**Fig. 3:**
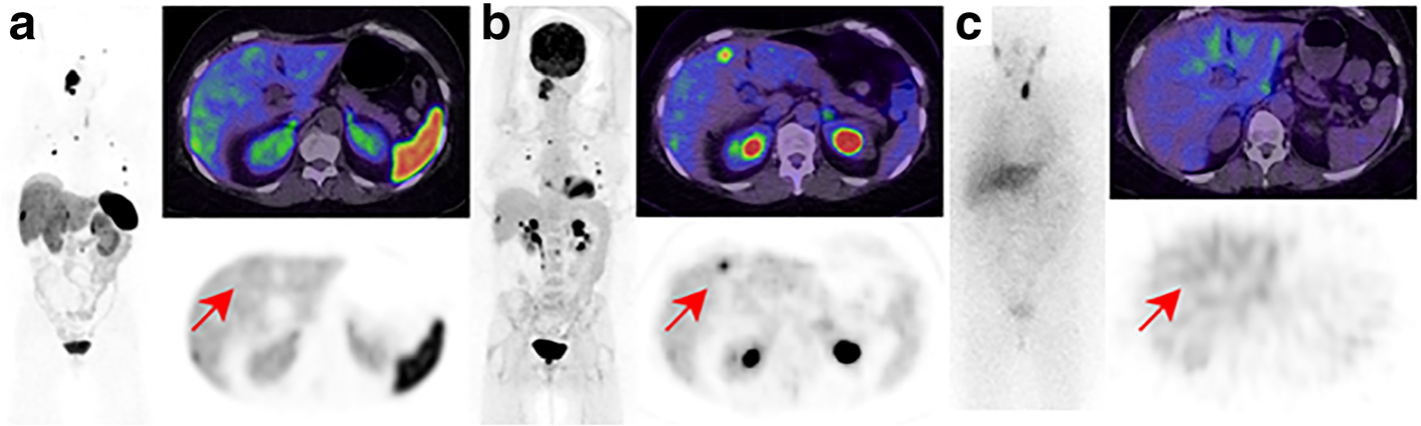
65 year-old female with negative germline mutation testing. The patient had a right glomus vagale with metastatic disease to lymph nodes, bone and liver evident on Ga-68 DOTATATE (**a**), F-18 FDG (**b**) but not I-123 MIBG (**c**). One of the hepatic metastases, however, was seen on FDG but not on other imaging modalities (*arrow*). The lower physiologic background activity on FDG may result in increased detection rate compared to DOTATATE in some patients

**Fig. 4:**
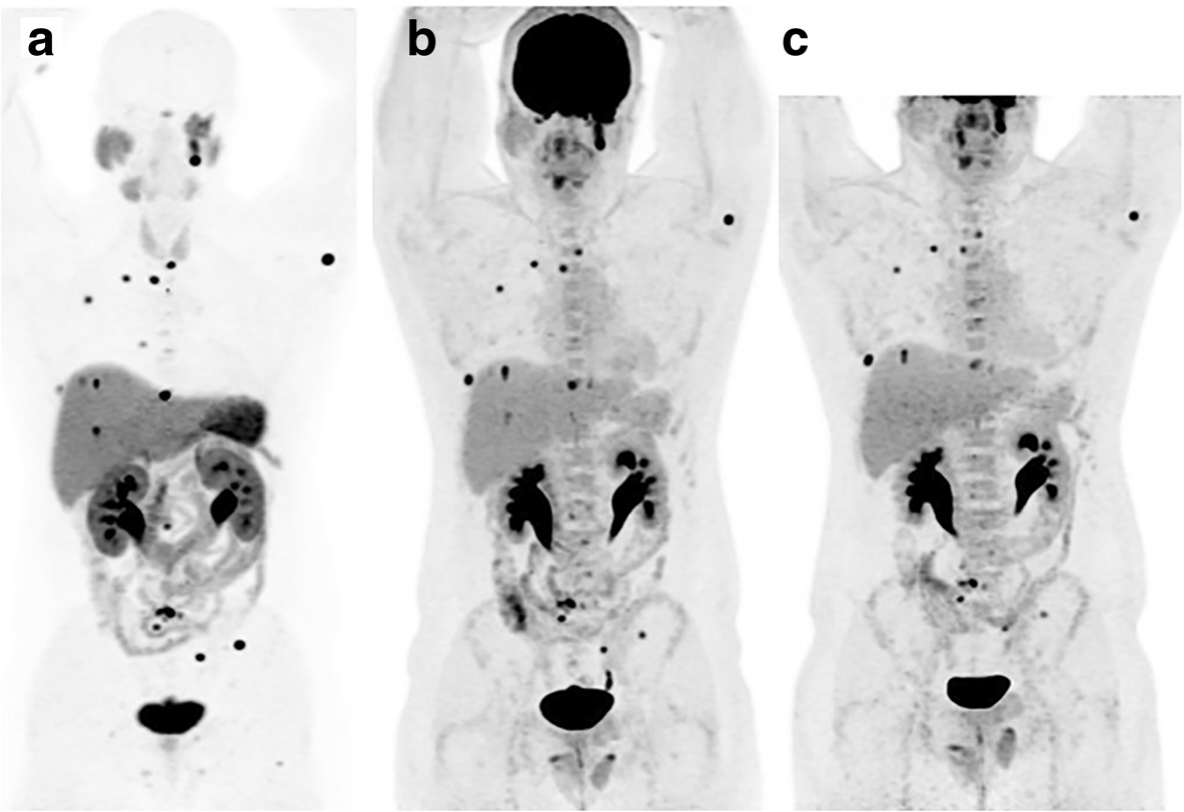
A 20 year old male with *SDHB* germline mutation and metastatic PCC. Baseline Ga-68 DOTATATE (**a**) and F-18 FDG (**b**) demonstrated metastatic disease with a concordant pattern of uptake. The patient was treated with 3 cycles of peptide receptor radionuclide therapy for uncontrolled hypertension. Follow-up DOTATATE and FDG imaging over a 2 year period demonstrated stable findings (**c**: FDG at 2 years). The high FDG-uptake was not predictive of an aggressive phenotype and reflects changes secondary to *SDHB* mutation and consequent disordered oxidative phosphorylation

FDG was positive in 95 of 104 lesions (91 %), with SUVmax ranging from 2 to 44, median 12.5 which was significantly less intense than 21 for DOTATATE PET/CT (*p* < 0.0001). Qualitatively, most lesions identified demonstrated moderate-to-high uptake. There were two patients with metastatic PGL and PCC respectively who had liver metastases not detected on DOTATATE. In both patients, the liver lesions were positive on FDG and negative on MIBG (Figs. 3 and 4).

**Fig. 5:**
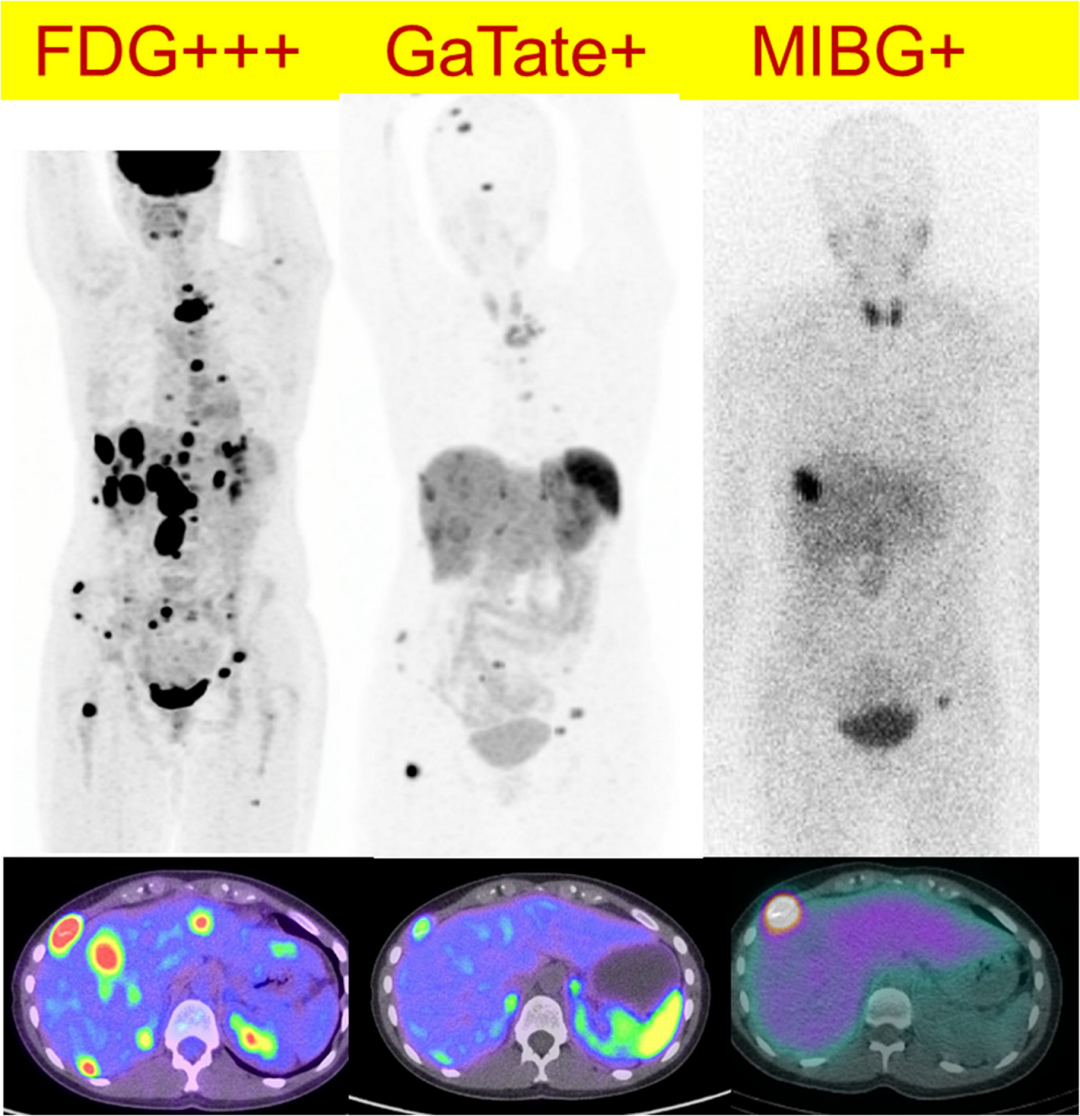
A 25 year old female with *SDHB* germline mutation and metastatic PGL. Sites of disease show very high F-18 FDG uptake (*left*) with relative low Ga-68 DOTATATE uptake (*middle*) and MIBG (*right*). The findings indicated lack of suitability for either radionuclide therapy with radiolabelled DOTATATE or MIBG. The patient had a rapidly progressive disease course despite treatment with chemotherapy (combination of cyclophosphamide, vincristine, dacarbazine) and subsequently sunitinib. The lack high SSTR expression is unusual for a *SDHB* phenotype and was indicative of de-differentiated disease with consequent aggressive phenotype

**Fig. 6:**
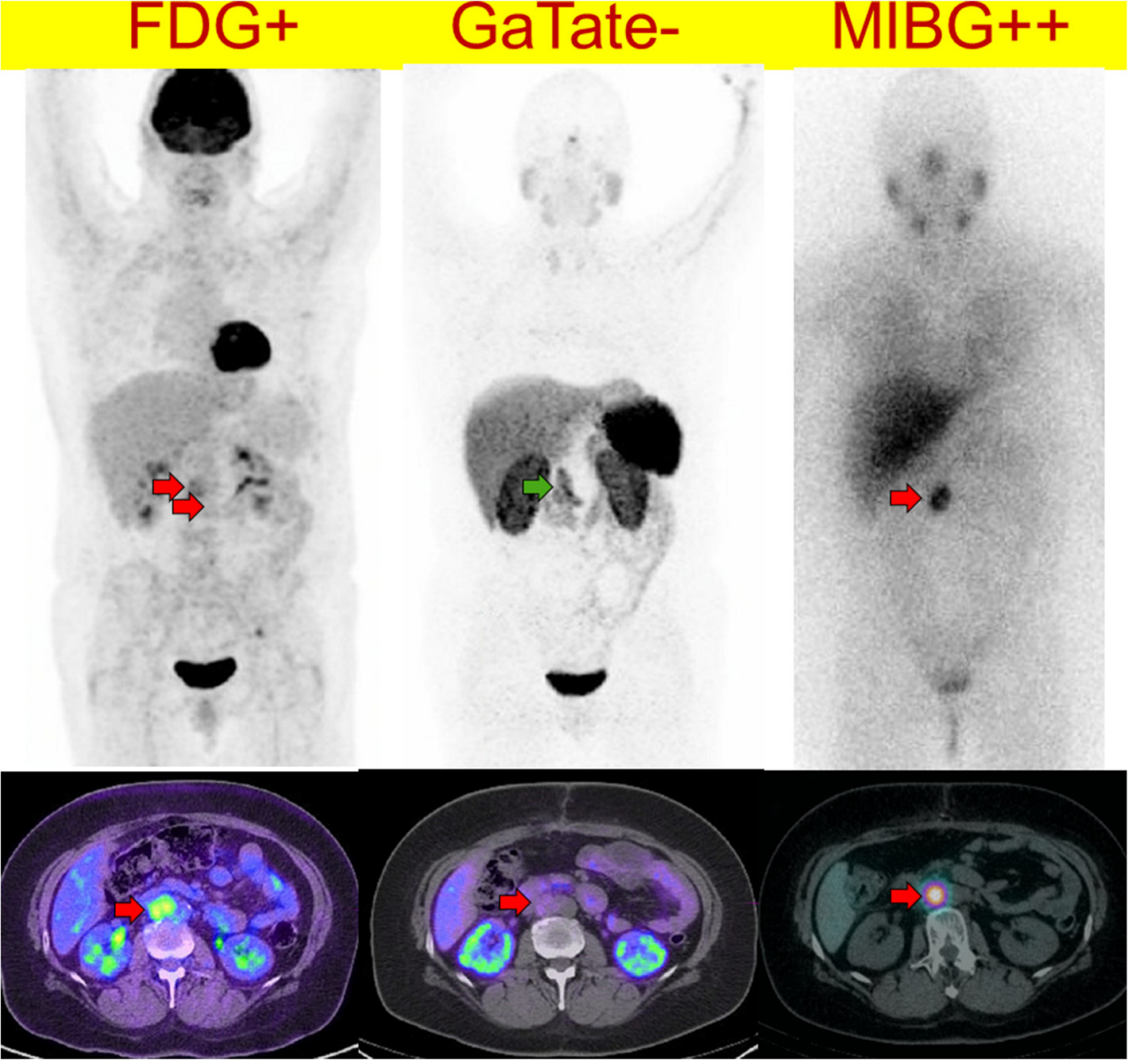
This middle-age female presented with back pain and a retroperitoneal mass on CT. F-18 FDG demonstrate moderate uptake (*left*), Ga-68 DOTATATE was negative (*middle*) with only physiologic uptake in the pancreas uncinate process (*green arrow*) while I-123 MIBG (*right*) demonstrated intense uptake. A paraganglioma was confirmed following surgical resection. Plasma and urine normetanephrine were elevated at 3728 pmol/L (*N* < 900) and 11.8 micromol/day (normal <2.3), respectively

Patients who had the aggressive phenotype of disease had higher F-18 FDG SUVmax (median 20.5) compared to patients with indolent or slowly progressive disease (SUVmax 10.0) (*p* < 0.001). However, four of these patients had an underlying SDHB germline mutation, while two had negative testing and one was not tested. A further four patients had very high FDG uptake similar to patients with aggressive disease but had stable disease with 1–3 year follow-up. Three of these patients had SDHB germline mutation suggesting metabolic reprogramming (Fig. 5). The combined findings of FDG and DOTATATE, however, may assist in disease characterisation with high FDG but low DOTATATE uptake being a potential indicator of aggressive disease (Fig. 6).

### I-123 and I-124 MIBG findings

MIBG was positive in substantially fewer lesions (17/56, 30 %). When positive, the majority of lesions demonstrated moderate-to-intense MIBG uptake. The only patient in our study who had I-124 MIBG PET/CT as part of workup for I-131 MIBG therapy was positive in 4 out of 8 of the lesions (50 %). There may, however, be a selection bias related to the high proportion of patients presenting with metastatic disease or known SDHB mutation. Additionally, as a tertiary oncology referral centre, patients with strongly positive MIBG imaging, especially in a primary lesion would be unlikely to be referred for DOTATATE scanning.

### Influence of catecholamine levels and beta-blockade on FDG PET/CT imaging appearances

Twelve patients had documented elevated serum or urinary catecholamines within six months of the time of imaging with FDG PET/CT (Table 3). Five patients had increased BAT uptake, which was severe in three cases with intensely avid BAT uptake above and below the diaphragm (Fig. 6). Two patients concurrently treated with beta (and alpha) blockade had no visible BAT uptake. However, half of patients treated with alpha blockade alone had visible BAT uptake.

**Table 3:** Clinical, biochemical and BAT FDG uptake characteristics of patients with elevated catecholamine levels

Age	Gender	Adr	NorAdr	Dop	BAT severity	SUVmax	Beta-blocker	Alpha-blocker
46	M	0.32	**20**	0.15	**Severe**	**19**	No	No
23	M	0.63	**15**	**1.1**	**Severe**	**18**	No	No
50	F	0.67	**8.2**	0.55	**Severe**	**10**	No	**Yes**
22	F	0.02	**7.5**	0.54	Nil	<2.5	No	**Yes**
30	M	0.84	**6.3**	0.60	**Mild**	**4.8**	No	**Yes**
26	F	0.22	**5.2**	N/A	Nil	<2.5	No	**Yes**
50	F	0.47	**4.1**	0.82	Nil	<2.5	**Yes**	**Yes**
74	M	**3.4**	**3.6**	0.16	Nil	<2.5	**Yes**	**Yes**
32	F	0.30	**3.1**	**1.2**	**Mild**	**5**	No	No
68	F	0.21	**2.4**	N/A	Nil	<2.5	No	No
21	M	0.28	**2.2**	0.86	Nil	<2.5	No	No
31	F	**1.7**	**1.3**	N/A	Nil	<2.5	No	**Yes**

*Adr* adrenaline, *NorAdr* noradrenaline, *Dop* dopamine, expressed as multiples of upper limit of reference range, *BAT* brown adipose tissue, *SUVmax* maximuma standardised uptake value, *N/A* not available. Items abnormal are highlight in bold

**Fig. 7:**
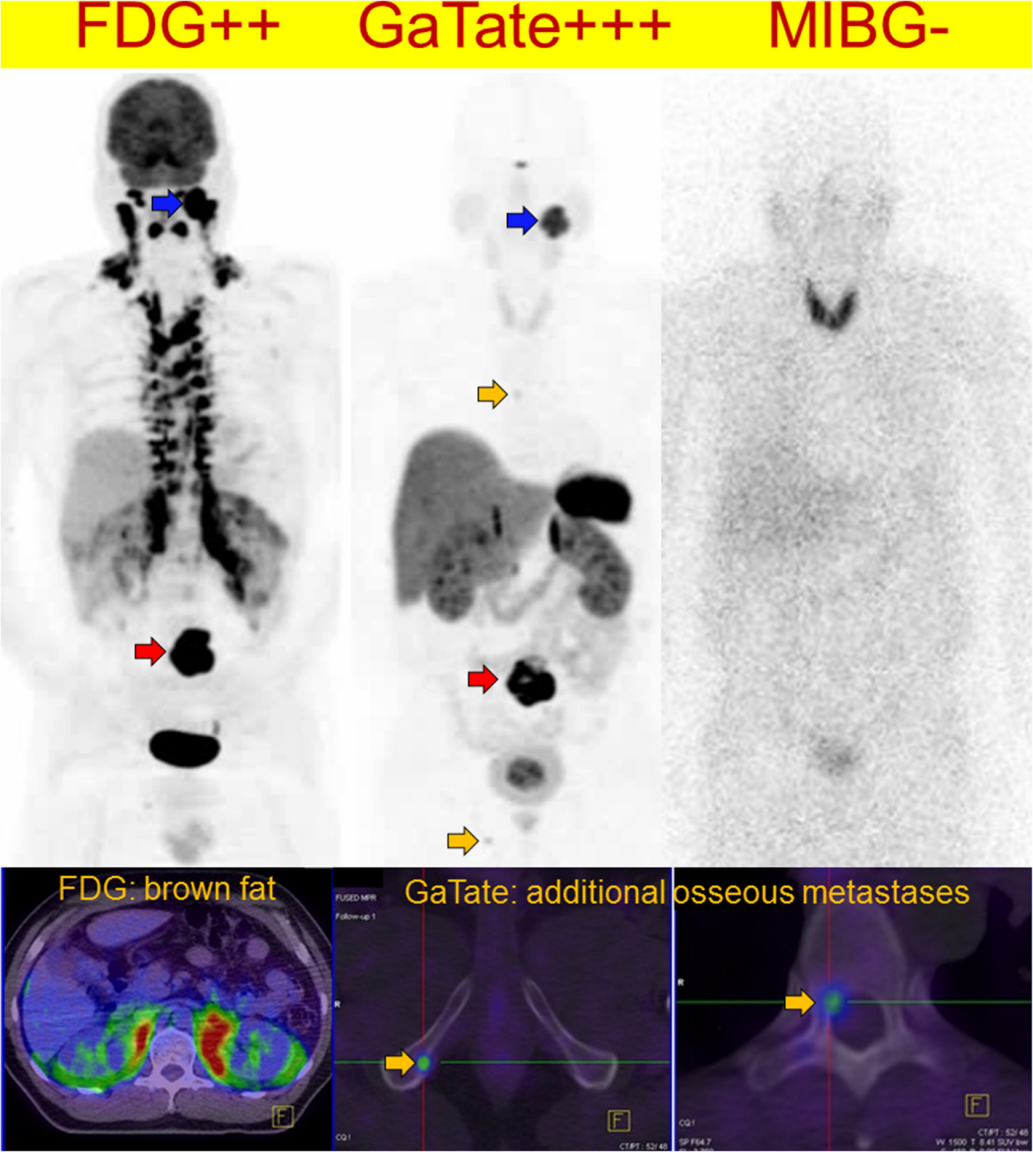
Patients with *SDHB*-related metastatic PGL showing extensive brown adipose tissue above and below the diaphragm on F-18 FDG PET/CT due to high circulating catecholamines (serum normetadrenaline 18 000 pmol/L, normal <900). F-18 FDG MIP (*left*) and axial fused PET/CT (*bottom*) and MIBG (*right*). The peri-nephric and, to a lesser extent central para-vertebral distribution is characteristic of PCC/PGL-related brown fat activation. Ga-68 DOTATATE (*middle*) clearly demonstrated an Organ of Zuckerkandl primary and several osseous metastases (*botttom middle* and *bottom right*), and is not subject to this physiologic artefact. As well as masking potential primary tumour sites along the sympathetic chain, the “sink effect” in brown fat limits availability of tracer for uptake in tumour sites

**Fig. 8:**
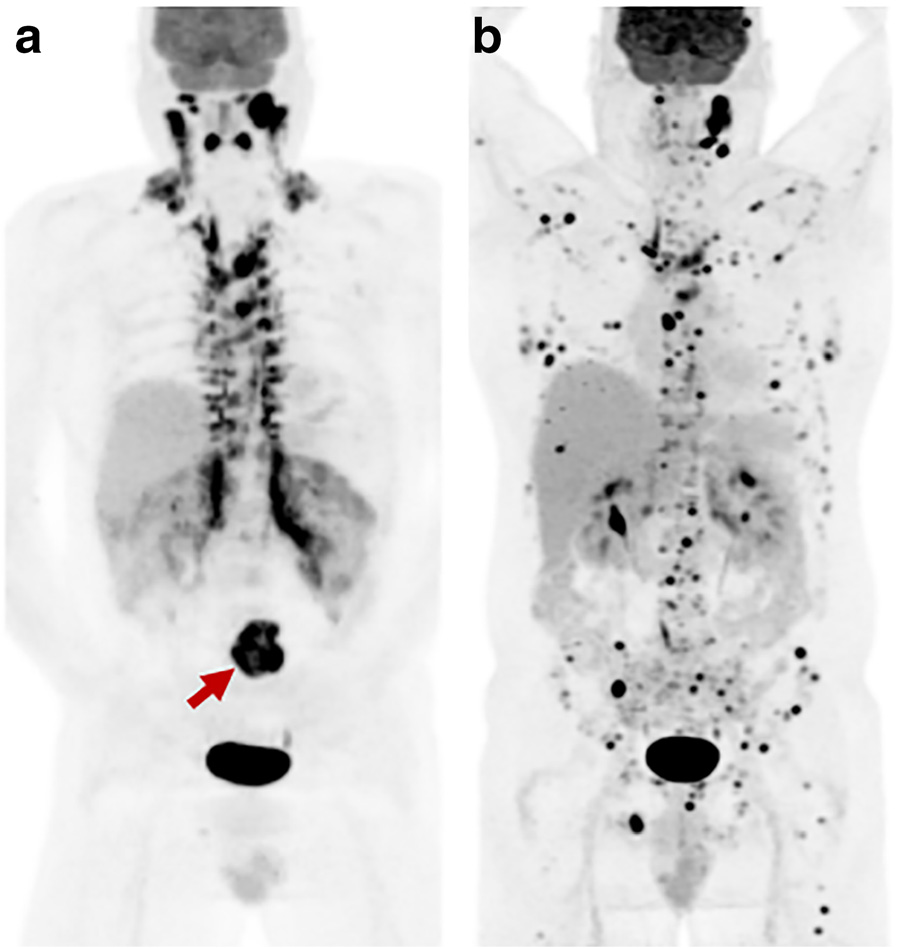
The same patient from Fig. 7 with baseline F-18 FDG (**a**) shown prior to commencement of alpha/beta-blockade. Following excision of the primary site (*red arrow*) there was marked progression of metastatic disease on FDG (**b**) with concordant biochemical progression. Despite rising normetadrenaline levels (>100,000pmol/L compared to 18,000pmol/L at baseline) brown fat activation is not visualised following commencement of alpha/beta-blockade

Two of the three patients with severely increased BAT uptake at baseline had follow-up FDG PET/CT imaging. One patient had interval treatment with alpha/beta-blockade and despite disease progression demonstrated near complete resolution of BAT (Figs. 7 and 8). A second patient had interval resection of four abdominal PGLs, normalisation of plasma normetadrenaline and complete resolution of BAT uptake. The BAT uptake was most intense and extensive in those patients with most elevated noradrenaline levels; all patients with noradrenaline levels more than 8 times the upper limit of normal (ULN) range had severe BAT uptake, whilst there was variable BAT uptake in those patients with noradrenaline levels 3–8 times ULN range. One patient had moderate BAT uptake despite contemporaneous normal plasma catecholamine levels, potentially reflecting underlying physiologic BAT activity.

## Discussion

In our cohort of patients, PGL and PCCs generally demonstrated high uptake on both DOTATATE and FDG PET/CT, although the intensity of uptake was significantly greater with DOTATATE PET/CT. Combined with lower background activity, this resulted in significantly superior tumour contrast with DOTATATE allowing easier and more confident diagnosis. Whilst this finding was largely true regardless of location (head and neck versus abdomen) or sporadic versus inherited cases, exceptions were found. The only site where DOTATATE had lower detection rate of lesions was in the liver, most likely due to the high background hepatic parenchymal uptake obscuring sites of disease. The specificity in diagnosing neuroendocrine tumours is also high for DOTATATE PET/CT [20], which overexpress SSR like PGL and PCCs with consequent high management impact [21]. Theranostic properties of DOTATATE also enables selection of patients potentially suitability for PRRT with either Lu-177 or Y-90 labelled DOTATATE [22, 23]. Further, DOTATATE can be used as a response assessment in this setting or follow-up in patients who are undergoing treatment and does not suffer from interactions with inhibitory medications compared to MIBG studies. Detection rate of DOTATATE is also much higher compared to the subgroup of our patients who had MIBG, the historical molecular imaging gold standard. This is consistent with prior series but may also partially reflect a selection bias in this series as detailed above.

Marked overexpression of SSTR-2 cell surface expression in PGLs and PCCs was described over two decades ago [11, 24]. It is therefore not surprising that DOTATATE, which has a predominant affinity for SSTR-2, is sensitive for imaging these tumours. Janssen et al. recently demonstrated significantly superior detection rate of DOTATATE PET/CT in a prospective series of 17 patients with SDHB-related metastatic PGLs and PCCs compared to FDG, fluorodihydroxyphenylalanine (FDOPA), fluorodopamine (FDA) and CT/MRI [25]. In particular, a lesion-based detection rate of 99 % was seen with DOTATATE compared to 86 % for FDG PET/CT. The same group has also demonstrated superior sensitivity of DOTATATE in detection of head and neck paragangliomas, especially SDHD-related tumours [26].

The higher lesional metabolic activity seen on FDG PET/CT in cases with mutations from the pseudohypoxic cluster (notably including SDH germline mutations) parallels the underlying biology and highlights the ability of molecular imaging to visualise consequences of mitochondrial mutations that result in altered glycolytic metabolism. Succinate dehydrogenase (SDH) is a mitochondrial membrane bound enzyme complex that catalyzes the oxidation of succinate to fumarate in the tricarboxylic acid cycle (TCA cycle). Loss of SDH enzyme activity leads to constitutive activation of hypoxia inducible factors (HIF) regardless of oxygen levels with resultant inhibition of oxidative phosphorylation and induction of less efficient glycolytic pathways consistent with the Warburg effect. The resultant metabolic reprogramming leads to very intense FDG uptake may occur despite a benign differentiated phenotype [27].

Our series includes some patients with SDHx-related mutations that had lower DOTATATE uptake in more aggressive cases presumably due to dedifferentiation and loss of SSTR-expression. Lower uptake intensity similar to or less than liver, however, was still clearly sufficient to detect sites of disease, with the exception of hepatic metastases. As expected, high FDG uptake is seen in aggressive metastatic disease [14, 27] regardless of germline mutation status and this was the case in several patients who succumbed to the disease shortly after diagnosis. Although SDHB germline mutation carriers have a higher risk of malignant degeneration [28–30], the intense FDG avidity is also evident in benign lesions and does not necessarily indicate aggressive or dedifferentiated disease [27]. This was the case in 3 of our patients who had stable burden of disease albeit metastatic, and did not have symptomatic or biochemical progression with a period of 1 to 4 years follow-up. Two patients, however, were treated with peptide receptor radionuclide therapy potentially confounding this observation. In the patient with SDHA germline mutation, the disease progressed very slowly over 5 years despite no treatment apart from local surgery and radiotherapy to the primary right glomus jugulo-tympanicum at presentation. Thus, for certain subtypes of PCC/PGLs with disordered oxidative phosphorylation, FDG alone cannot characterise the aggressiveness of disease. The combination of low DOTATATE and high FDG uptake in these subtypes, however, may represent a specific feature of aggressive disease. The variation of uptake we have observed is summarised in Table 4 and suggested key indications in Table 5.

**Table 4:** Variable patterns of uptake by PCC/PGL subtype

	Examples	SSTR PET	FDG PET	MIBG SPECT/PET
Pseudohypoxic	SDHx, VHL, HIF2	+++ ^a^	+++	- (40 %) to ++
Kinase signalling	RET (MEN2), NF1, TMEM127, MAX	+ to ++	- or +	++ to +++

^a^may diminish in small subgroup of patients with aggressive phenotype

**Table 5:** Key indications for molecular imaging in PCC/PGL

▪ Diagnosis and localisation of adrenal-and extra-adrenal PCC/PGL in patients with elevated catecholamine levels.
▪ Staging of patients prior to surgical resection to exclude metastatic or additional primary sites of disease.
▪ Selection of patients for radionuclide therapy with ^177^Lu/^90^Y radiolabelled DOTATATE or ^131^I-MIBG therapy.
▪ Response assessment following chemotherapy or targeted therapies.
▪ Surveillance of patients with inherited mutations such as SDHxor VHL

Compared to conventional molecular imaging with I-123 MIBG, several authors have suggested the better detection rate of DOTATATE [31, 32] and FDG [15, 27] PET/CT and our results largely confirm these findings. This in part is due to higher spatial resolution of PET/CT compared to SPECT/CT. Even in the case of I-124 MIBG imaging in one of our patients, the number of lesions detected was lower compared to both DOTATATE and FDG PET/CT. MIBG however, remains an essential modality for selection of patients for MIBG therapy.

Integration of the molecular imaging phenotype into patient management is complementary to genetic testing and histopathology, providing disease characterisation on a whole body scale [33]. Molecular imaging phenotype may suggest the type of mutation and direct genetic testing as mutational status is often not known at the time of imaging. In our experience, either DOTATATE or FDG PET/CT can be utilised for diagnosis in patients with suspected PGL/PCC when mutation status is not known but DOTATATE being theranostic is far more suitable for our patient population. Several other radiopharmaceuticals are also used to image PGL/PCC such as F-18 FDOPA [34], F-18 FDA and C-11 HED [35, 36]. Because our patient population is mostly referred to us for diagnosis and consideration for PRRT or I-131 MIBG therapy, the non-theranostic properties are major drawbacks and some radiopharmaceuticals are either not readily available or have not been approved for clinical use in Australia.

Several cases demonstrated extensive activation of BAT on FDG due to very high circulating catecholamines, with follow-up imaging demonstrating resolution of BAT activity in two cases after successful treatment with surgery, PRRT and alpha blockade. BAT activation is commonly stimulated by cold exposure and inversely correlated with body mass index, although it is also a less appreciated but important characteristic of catecholamine secreting PGL/PCCs [37, 38]. Given that beta-blockers are often administered to suppress BAT uptake for repeat imaging [39], it is critical to consider the possibility of PGL/PCC in the appropriate clinical context (e.g., assessment of adrenal/paravertebral lesion) because isolated beta-blockade may precipitate hypertensive crisis due to unopposed alpha-adrenergic activation. The absence of brown fat activation in several cases despite documented high catecholamine levels is likely explained by concurrent treatment with alpha/beta blockers. A recent Chinese study [40] comparing BAT uptake on FDG PET/CT in patients with PGL/PCC and normal controls demonstrated a positive correlation between total metanephrine levels and BAT FDG avidity is consistent with our series.

There are several limitations to this study. This was a retrospective analysis and a relatively small patient population, albeit acknowledging this is a rare disease. There is also patient selection bias as our institution performs peptide receptor radionuclide therapy resulting in a higher proportion of patients with metastatic disease and our results may not be generalizable to patients with non-metastatic PGL/PCC. We also did not have patients with known VHL, TMEM127, NF1 and MAX germline mutations, again acknowledging the rarity of some of these mutations. Secondly, not all our patients had MIBG studies to enable better comparison of newer molecular imaging techniques to the current molecular imaging gold standard in PGL and PCC imaging. Thirdly, for ethical reasons, histopathology was not performed on all lesions to confirm the reasons for apparent differences in DOTATATE, FDG and MIBG uptake.

## Conclusion

Acknowledging the heterogenous imaging nature of PGL and PCCs, our series suggests DOTATATE PET/CT is a suitable first-line hybrid molecular and anatomic study due to its theranostic properties, superior tumour contrast compared to FDG PET/CT and vastly superior detection rate compared to MIBG planar and SPECT/CT. Ease of preparation, lack of confounding brown fat activation and patient-friendly imaging are additional advantages. Performing a staging DOTATATE PET/CT allows clinicians to confidently diagnose or exclude the disease, guide future management, further investigations particularly germline mutation testing and avoid unnecessary procedures or biopsies [41]. MIBG should generally only be performed if I-131 MIBG therapy is being considered and FDG PET/CT provides additional information regarding the possibility of pseudohypoxia cluster germline mutation, metabolic disease aggressiveness and directing sites for histologic analysis if required.

## Abbreviations

FDG: ^18^F-fluorodeoxyglucose
MIBG: Metaiodobenzylguanidine
MIP: Maximum intensity projection
PCC: Pheochromocytomas
PGL: Paragangliomas
SUV: Standardised uptake value
